# Conotoxins as Tools to Understand the Physiological Function of Voltage-Gated Calcium (Ca_V_) Channels

**DOI:** 10.3390/md15100313

**Published:** 2017-10-13

**Authors:** David Ramírez, Wendy Gonzalez, Rafael A. Fissore, Ingrid Carvacho

**Affiliations:** 1 Centro de Bioinformática y Simulación Molecular, Universidad de Talca, 3460000 Talca, Chile; davramirez@utalca.cl (D.R.); wgonzalez@utalca.cl (W.G.); 2 Instituto de Ciencias Biomédicas, Universidad Autónoma de Chile, 3460000 Talca, Chile; 3 Millennium Nucleus of Ion Channels-Associated Diseases (MiNICAD), Universidad de Talca, 3460000 Talca, Chile; 4 Department of Veterinary and Animal Sciences, University of Massachusetts, Amherst, MA 01003, USA; rfissore@umass.edu; 5 Department of Biology and Chemistry, Faculty of Basic Sciences, Universidad Católica del Maule, 3480112 Talca, Chile

**Keywords:** conotoxins, voltage-gated calcium (Ca_V_) channels, ω-conotoxin structure, therapeutic potential

## Abstract

Voltage-gated calcium (Ca_V_) channels are widely expressed and are essential for the completion of multiple physiological processes. Close regulation of their activity by specific inhibitors and agonists become fundamental to understand their role in cellular homeostasis as well as in human tissues and organs. Ca_V_ channels are divided into two groups depending on the membrane potential required to activate them: High-voltage activated (HVA, Ca_V_1.1–1.4; Ca_V_2.1–2.3) and Low-voltage activated (LVA, Ca_V_3.1–3.3). HVA channels are highly expressed in brain (neurons), heart, and adrenal medulla (chromaffin cells), among others, and are also classified into subtypes which can be distinguished using pharmacological approaches. Cone snails are marine gastropods that capture their prey by injecting venom, “conopeptides”, which cause paralysis in a few seconds. A subset of conopeptides called conotoxins are relatively small polypeptides, rich in disulfide bonds, that target ion channels, transporters and receptors localized at the neuromuscular system of the animal target. In this review, we describe the structure and properties of conotoxins that selectively block HVA calcium channels. We compare their potency on several HVA channel subtypes, emphasizing neuronal calcium channels. Lastly, we analyze recent advances in the therapeutic use of conotoxins for medical treatments.

## 1. Introduction

Venomous cone snails (*Conus*) produce several toxic peptides, conopeptides, which target the neuromuscular system of their prey, worms, mollusks, snails and fishes [1,2]. Conotoxins are peptides of 20–30 residues whose main structural characteristic is a rigid backbone formed by disulfide bonds between six cysteines. Conotoxins can be classified according to several criteria, including: *a.* the gene superfamily they belong to; *b.* the pattern of cysteine distribution, cysteine framework; and *c.* their molecular targets. Table 1 summarizes the known groups of conotoxins and their protein targets.

The toxins produced by the genus *Conus* are numerous and diverse, and approximately 6200 different toxins have been isolated and identified from more than 100 different species thus far [14,15]. The target of most of these toxins are ion channels, including voltage- and ligand-gated channels, as well as G-protein coupled receptors [16,17]. In this review, we will focus on ω-conotoxins, which modulate Ca_V_2.X channels. ω-conotoxins prevent entry of calcium (Ca^2+^) through these voltage-activated Ca_V_, channels at the presynaptic nerve terminal, thereby, interfering with the release of vesicles containing acetylcholine and neurotransmission [13]. In general, ω-conotoxins impede Ca^2+^ flux by physically occluding the channel pore [18]. The kinetics of the binding is variable and can show slow dissociation rates, generating poorly reversible blockage and long term inhibition [18].

### 1.1. Voltage-Gated Calcium Channels

Voltage-gated Ca^2+^ (Ca_V_) channels are transmembrane proteins that belong to the same transmembrane gene superfamily as the Na_V_ and the K_V_ channels. Ca_V_ channels can be organized into two groups according to the voltage changes required for activation: Ca^2+^ channels that require “larger” depolarizations to be opened (when compared with the current-voltage relation for *I_Na_*) are known as high-voltage activated (HVA) channels, whereas Ca^2+^ channels that open at more negative potentials are known as low-voltage activated (LVA) [19]. Ca_V_s are composed of a pore forming subunit, α_1_, encoded by the CACNA1x genes (see Table 2). L-Type Ca_V_s, Ca_V_1.1–1.4, are known as α_1_S, α_1_C, α_1_D, and α_1_F. The P/Q-, N- and R-type, Ca_V_2.1–Ca_V_2.3, are termed as α_1_A, α_1_B, and α_1_E. Finally, the T-Type, Ca_V_3, are composed of α_1_G, α_1_H, and α_1_I (Table 2 [20,21]). Depolarizations provoked by the opening of Ca_V_ channels shape the action potential in the heart, regulate muscle contraction, and modulate neurotransmitter secretion at nerve terminals. In general, “*excitable cells translate their electricity into action by Ca^2+^ fluxes modulated by voltage-sensitive, Ca^2+^-permeable channels*” [19]. Once Ca^2+^ ions gain access to the cytosol, they act as second messengers, capable of binding thousands of proteins affecting their localization and function. Variations of intracellular Ca^2+^ concentrations influence many cell functions such as transcription, motility, apoptosis and initiation of development [22].

The expression and properties of the pore forming α subunit are modified by two main auxiliary or accessory subunits: α_2_δ and β, which regulate the channel’s biophysical properties, its trafficking, and membrane expression. Ca_V_1 and Ca_V_2 channels can form heteromeric complexes co-assembling with different α_2_δ subunits, which are encoded by *CACNA2D1-4* genes, and β subunits, which are encoded by *CACNB1-4* genes. The stoichiometry of this assembly is of one β subunit and one α_2_δ accessory subunit. An additional accessory subunit, γ, has been reported only in skeletal muscle [20].

### 1.2. Ca_V_2.X Channels

The channels of the Ca_V_2 family is formed by a pore-forming Ca_V_α1 subunit plus the auxiliary subunits Ca_V_β and Ca_V_α_2_δ, with the Ca_V_α1 subunit defining the channel subtype, as shown previously (see Table 2). The Ca_V_2.1 channels conduct currents classified as P-type and Q-type that are well described in neurons, whereas the Ca_V_2.2 and Ca_V_2.3 channels underpin the N-type and R-type currents, respectively, also characterized in neurons [23].

Ca_v_2 channels are responsible for the Ca^2+^ influx required for the fast release of neurotransmitters as well as for the release of hormones from secretory-type cells such as chromaffin cells [24]. Ca_V_2 channels also regulate neuronal excitability via activation of the Ca^2+^ activated K^+^ channels that in turn control repolarization and hyperpolarization [25]. Consistent with these functions, Ca_V_2.1 null mice exhibit ataxia and die around 4 weeks after birth [26]. Mice deficient in Ca_V_2.2 channels, N-type, showed suppressed response to pain, which is consistent with the use of conotoxins as analgesics [27], and with the expression of Ca_V_2.2 channels in nerve terminals in association with pain receptors. Ca_V_2.2 channels are involved in neurotransmitter release of nociceptive pathways from afferent terminals in the ventral and dorsal horn of the spinal cord and dorsal root ganglion neurons [20,28]. Ca_V_2.3 null mice also show reduced pain sensitivity [23].

### 1.3. General Properties of ω-Conotoxins

**Figure 1:**
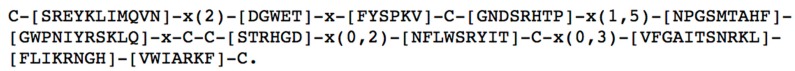
ω-Conotoxins family pattern (PROSITE ID: PS60004). The pattern is described using the following conventions: ‘x’ is used for a position where any amino acid is accepted; ambiguities are indicated by listing the acceptable amino acids for a given position, between square parentheses ‘[ ]’, i.e., [ALT] stands for Ala or Leu or Thr. Each element in the pattern is separated from its neighbor by a ‘-‘. Repetition of a pattern element can be indicated by following that element with a numerical value or a numerical range in brackets. Examples: x(2) corresponds to *x-x*, x(1,5) corresponds to *x* or *x-x* or *x-x-x* or *x-x-x-x* or *x-x-x-x-x*.

ω-conotoxins are small peptides ranging in size from 13 to 30 amino acids. They have net charges between +5 and +7 [17], are mostly polar and are highly water soluble. They show three disulfide bridges that are formed between conserved cysteine residues that are arranged in the following organization, C-C-CC-C-C [29]; they form a common structural motif consisting of a cysteine knot, which is also present in toxic and inhibitory polypeptides [30]. The ω-conotoxins family exhibit a characteristic pattern signature described in the PROSITE database [31,32] (see Figure 1). 

Most ω-conotoxins characterized to date are selective for N-type Ca_V_ channels. As indicated, the main mechanism of action of ω-conotoxins’ is by blocking the channel pore [33], which is accomplished by tight binding of the toxin to the channel pore [18]. The most studied and defined ω-conotoxin is GVIA isolated from *Conus geographus* [34]. Its specific activity against N-type Ca^2+^ channels—Ca_V_2.2 channels- [35] was established in neuronal cell types [36]. Other ω-conotoxins from the venom of different Conus species include CVID from the venom of *Conus catus*, CNVIIA from *Conus consors* and MVIIA, MVIIIV, and MVIID from *Conus magus* have been identified [37]. Additional ω-conotoxins have been isolated from other *Conus* such as *striatus* [38], and *magus* [39].

## 2. Classification of ω-Conotoxins That Target Ca_V_ Channels

### 2.1. C. geographus—GVIA

**GVIA.** It consists of 27 amino acids with a backbone constrained by the formation of three disulfide bonds (Cys^1^–Cys^16^, Cys^9^–Cys^20^, and Cys^15^–Cys^26^). The possible toxic effect of GVIA and of the other members of the family such as GVIB, GVIC, GVIIA, and GVIIB was determined by performing intracerebral injections in mice, which provoked involuntary movements (“shaking”) in the animals [34] (Table 3). In vitro studies were first performed on nerve-muscle preparations of frogs where GVIA irreversible blocked the voltage-activated Ca^2+^ channels of the presynaptic terminal preventing acetylcholine exocytosis [13]. Together, these studies showed that GVIA selectively inhibits Ca_V_2.2 channels in an irreversible manner. The site of action of GVIA on Ca_V_2.2 was found to be on the large extracellular domain III between the S5–S6 trans-membrane regions [18]; mutagenesis studies further showed that the reversibility of the block induced by GVIA and MVIIA was dramatically enhanced by swapping a glycine residue at position 1326 for a proline. GVIA also binds the α1 subunit of the Torpedo nAChR [13]. The 3D structures of GVIA resolved by NMR spectroscopy deposited in the Protein Data Bank (PDB) are: 2CCO [40], 1TTL [41], and 1OMC [42].

### 2.2. C. magus—MVIIA and MVIIC

**Figure 2:**
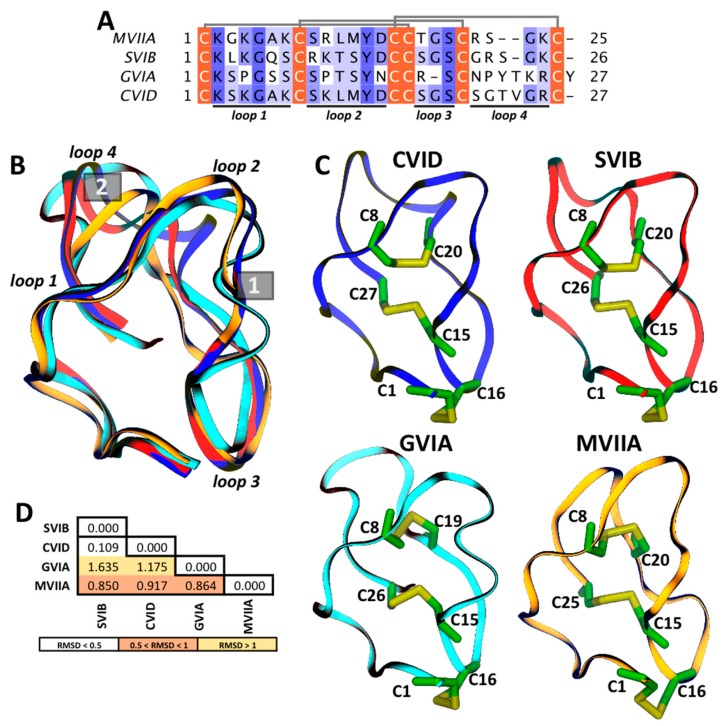
Structural differences between ω-conotoxins. (**A**) Multiple sequence alignment of ω-conotoxins colored by conservation in a ramp, from white (not conserved) to dark blue (highly conserved); cysteines involved in disulfide bonds (gray lines) are highlighted in orange, and loops are indicated at the bottom; (**B**) Structural alignment of CVID (model from Swiss Model Repository ID: P58920); SVIB (PDB ID: 1MVJ); GVIA (PDB ID: 2CCO) and MVIIA (PDB ID: 1MVI); the ω-conotoxins backbone is represented as a ribbon diagram. Major structural differences in ω-conotoxins are labeled as 1 and 2. (**C**) ω-conotoxins in ribbon representation with disulfide bonds in stick representation. (**D**) RMSD (Å) matrix from ω-conotoxins backbone atoms structural alignment.

**MVIIA.** Also known as ziconotide is a 25 amino acid peptide that also blocks the pore of Ca_V_2.2 channels (Table 3) and induced potent analgesia in rodents [43] and human patients with persistent cancer pain [44]. In December 2004, the Food and Drug Administration (FDA) approved Prialt^®^ (commercial name for MVIIA) for the treatment of severe chronic pain using an intrathecal pump system to deliver the drug into the cerebrospinal fluid. Consistent with this action, injection of MVIIA into mammals caused important neuromuscular effects such as decrease of spontaneous and coordinated locomotor activity and tremors [45]. It was shown that these effects and the pain relief caused by delivery of MVIIA into the cerebrospinal fluid are mediated by inhibition of the release of pro-nociceptive neurochemicals such as glutamate, calcitonin gene-related peptide (CGRP), and substance P into the brain and spinal cord [46,47]. Site-mutagenesis studies revealed that the Met^12^ residue in loop 2 (Figure 2) is the responsible for the toxicity of MVIIA. Met^12^ interacts with the hydrophobic pocket residues Ile^300^, Phe^302^, and Leu^305^, located between repeats II and III of Ca_V_2.2 channels; this interaction disrupts the normal function of the channel [45]. Systematic mutations of the residues in the loop 2 of MVIIA as well as of other ω-conotoxins may be used for future drug design to develop modulators of Ca_V_2.2 with lower side effects and higher effectiveness [45]. The 3D structures of MVIIA resolved by NMR spectroscopy deposited in the PDB are: 1OMG [48], 1MVI [49], 1TTK [50], 1DW4 [51], and 1DW5 [51].

**MVIIC.** This toxin blocks Ca_V_2.1 and Ca_V_2.2 channels (Table 3). It possesses similar characteristics to those described for MVIIA and its intracerebral injection in mice caused progressive decrease in respiration rates with marked signs of gasping for breath. The peptide was lethal at low doses (0.1–0.4 μg [52]). The 3D structures of MVIIC resolved by NMR spectroscopy deposited in the PDB are: 1OMN [53] and 1CNN [54].

### 2.3. C. striatus—SVIA and SVIB SO-3

**SVIA and SVIB.** The SVIA toxin contains 24 amino acids. Its administration into lower vertebrates such as fish and frogs provokes paralysis [38], although it has relatively poor activity against mammalian Ca^2+^ channels. While SVIA blocks only Ca_V_2.2, SVIB blocks P/Q type and N-type channels (Table 3). SVIB induces respiratory distress in mice when injected intracranially at concentrations of 70 pmol/g mouse and it is lethal around 300 pmol/g mouse; SVIA administration does not kill mice even at extremely high doses [38]. 

**SO-3.** This ω-conotoxin shows analgesic activity similar to that of MVIIA when tested in models of acute and chronic pain in rodents, however, it has fewer adverse effects than MVIIA [45,55]. The 3D structure of SO-3 resolved by NMR spectroscopy deposited in the PDBe is: 1FYG [56].

### 2.4. C. catus—CVID

**CVID.** The sequence of its loop 4 is less conserved than other of ω-conotoxins. It displays the highest selectivity for N-type over P/Q- type Ca^2+^ channels (radioligand binding assays) [57] and because of this it has been tested in clinical trials as analgesic [58]. The 3D of CVID structure resolved by NMR spectroscopy deposited in the PDB is: 1TT3 [50].

### 2.5. C. fulmen—FVIA

**FVIA.** It is reported to only be effective against Ca_V_2.2 channels [59]. The 3D structure of FVIA resolved by NMR spectroscopy deposited in the PDB is 2KM9 (to be published).

### 2.6. C. textile—TxVII and CNVIIA

**TxVII.** This conopeptide is very hydrophobic and has net negative charge of −3. The sequence of TxVII is 58% identical to that of δ-conotoxin-TxVIA, which targets Na^+^ channels. This toxin blocks the slowly inactivating, dihydropyridine- (DHP-) sensitive current [60]. The 3D of TxVII structure resolved by NMR spectroscopy deposited in the PDB is: 1F3K [61].

**CNVIIA.** This toxin is closely related to the CnVIIH toxin (Table 3), which possesses an unprocessed final glycine and therefore lacks amidation of its C-terminal end [62,63]. CNVIIA blocks Ca_V_2.2 channels but surprisingly it does not block the neuromuscular junction of amphibians. Intracerebroventricular injection of CNVIIA in mice causes shaking movements and mild tremors, depending on dosage, whereas when injected intramuscularly into fish it causes paralysis and death at higher doses [62].

## 3. Structural Characteristics of ω-Conotoxins and Blockade Site on the Ca_V_ Channels

ω-conotoxins share several structural characteristics that allow them to block multiple Ca_V_s on diverse cell types. Here we explore in detail four well known ω-conotoxins, CVID, SVIB, GVIA, and MVIIA, whose 3D structures have been resolved by NMR except for CVID (PDB IDs 1MVJ [49], 2CCO [40], and 1MVI [49], respectively); their multiple sequence alignments as well as their 3D structures are shown in Figure 2. As previously noted, they share four loops and three disulfide bonds (Figure 2A,C), giving them the same structural pattern (Figure 2B). These similarities are evident between CVID and SVIB (RMSD_backbone_ = 0.109 Å), although they are more subtle between CVID and GVIA (RMSD_backbone_ = 1.635 Å) (Figure 2D). The main structural differences between loops 2 and 3 (structural difference 1) and 4 (structural difference 2), where ω-conotoxins residues are not highly conserved, are highlighted with gray boxes (Figure 2B). Despite the structural similarities, there are differences in the selectivity of targets between these toxins. To understand the selectivity of these toxins at the structural basis, using NMR spectroscopy, researchers have determined the secondary and tertiary structures [50]. Adams et al. found a correlation between the solvent accessible surface area and the selectivity of ω-conotoxins, where the most exposed residue, R10 in MVIIA, play a crucial role in binding to Ca_V_s [50]. The residue(s) on Ca_V_ channels that interacts with ω-conotoxins is not yet elucidated, although the extracellular linker region between the P-region and S5 in domain III, the pore of Ca_V_2.2, is reported to be the area where the toxins bind channels [17,33]. In this region, G1326 appears to be the essential residue, as its mutation modifies the access of GVIA and MVIIA to the active site [69].

The structure-activity relationship (SAR) studies conducted in conotoxins identified key residues involved in the interaction with protein targets as well as identification of specific amino acids involved in their structural arrangement. These studies have been used to design small bioactive mimetics to selectively block Ca_V_2.2 over Ca_V_2.1 channels [70,71]. Bioactive mimetics have become promising candidates in the search for novel drugs for the treatment of chronic pain [21]. For example, based on the 3D structure of MVIIA [48] and identification of key residues such as K2, R10, L11, Y13, and R21 involved in the binding of MVIIA [72]. The data collected gave fundamental information for the design of the first bioactive mimetic of MVIIA in 1998, including the draft of small structures to mimic the residues R10, L11, and Y13 [73]. Although this bioactive mimetic showed poor inhibition against Ca_V_2.2 (19% at 10 μM), a second generation of mimetics was produced and two of these compounds showed promising activities against Ca_V_2.2 (IC_50_ = 3.3 and 2.7 μM) [74]. Since then, others ω-conotoxins mimetics have been reported using SAR information [70,75,76].

## 4. Therapeutic Uses of Conotoxins

The therapeutic and pharmacological potential of the conotoxins is well-known [1,47,77]. Nevertheless, their intrinsic physic-chemical and therapeutic characteristics such as molecular weight and low bioavailability due to their susceptibility to peptidase degradation has prevented the widespread use of conotoxins in the clinic. Importantly, and despite these limitations, their ability to selectively bind closely related molecular targets is an important strength of these marine conopeptides. Another advantage of Conotoxins is the diversity of targets, as they can act upon ion channels such as K_V_, Na_V,_ and Ca_V_ channels, as well as on several G-protein coupled receptors including neurotensin, α-adrenergic, and vasopressin receptors and also on ligand-gated receptors such as AChRs, 5HT3Rs, and NMDARs [77]. These properties make them excellent candidates to develop new bio-compounds and derivatives against pathologies such as pain, stroke, and convulsive disorders. Especially interesting is their specific affinity for N-type, Ca_V_2.2, Ca_V_ channels, which is a useful pharmacological characteristic for the validation of molecular targets, for example, in neuropathic pain. Ca_V_s channel-mediated cellular events can be modulated for therapeutic purposes by direct block of Ca_V_2.2, i.e., small peptides as conotoxins; by activation of GPCRs, or by direct interference with the channel trafficking [23].

In nature the proteins targeted by the cone snails on the preys are closely related to the proteins targeted in humans; however small structural and physiological differences can modify the efficacy, selectivity, and potency of conotoxins. Moreover, the target protein in cone snail’s preys may serve functions that are distinct to those in humans. Further, in humans and mammals the target proteins may be found in protected physiological spaces such as the Central Nervous System [77].

The recent significant progress in the identification of novel pharmacological targets for analgesic drugs designed using natural products has promoted the therapeutic use of conotoxins in pain relief. The main analgesic conopeptide is the ω-conotoxin MVIIA (Prialt^®^), which was approved for the management of severe chronic pain [43,47]. Prialt^®^ is being manufactured and labeled by Jazz Pharmaceuticals and Eisai Limited in the US and the European Union, respectively. Prialt^®^ blocks selectively N-type Ca_V_ channels through the inhibition of the presynaptic neurotransmitter release [13,78]. Prialt^®^ attenuates nociception in several animal models such as models of persistent pain [79], chronic inflammatory pain [80], neuropathic pain [81], and postoperative pain [43]. Prialt^®^ showed high effectiveness in morphine tolerant murine models [82], and prolonged Prialt^®^ intrathecal infusion does not produce tolerance to its analgesic effects [79,82]. Another ω-conotoxin with analgesic activity is CVID (AM336), a conopeptide selective for N-type Ca channels [83], although it might have greater side effects than MVIIA [83]. Other conotoxins used in analgesia are Contulakin-G (CGX-1160), MrIA (Xen-2174), Conantokin-G (CGX-1007), Vc1.1 (ACV-1), and MrVIB (CGX-1002) [77].

The pharmacological and therapeutically pre-clinical efficacy of MVIIA and CVID, along with the FDA approval of Prialt^®^, have established ω-conotoxins (and conotoxins in general) as viable platforms for the design of new and specific drugs to alleviate pain by aiming N-type Ca_V_ channels.

## 5. Conclusions

Neuronal Ca_V_ channels have potential as targets for treatments of pain and the selectivity of conotoxins for these channels render conopeptides valuable therapeutic tools. ω-conotoxins display an inhibitory cysteine knot which is also present in other toxic peptides. This motif, along with other common structural characteristics, is the basis of their potent and selective blocking activity on the pore of Ca_V_ channels. A ω-conotoxin, MVIIA, has been approved by the FDA for therapeutic use under the commercial name of Prialt^®^. Going forward, however, more widespread applications of conotoxins will require improvements to enhance their transport across the blood-brain barrier as well as modification to increase their chemical stability.

The association between the structure of ω-conotoxins and their activity against Ca_V_ channels remains undetermined and such knowledge will be fundamental to improve their use as therapeutic agents. Techniques such as circular dichroism and NMR spectroscopy have been helpful in the development of SAR studies, which have aided in the design of MVIIA [84], and GVIA [85] analogues. Additionally, the combination of electrophysiology, computational biophysics approaches, and SAR studies has provided new insights into the molecular binding mechanism of ω-conotoxins to their targets. This knowledge now places the drug design processes targeting chronic pain in a robust position to develop novel therapeutic agents. The design of small mimetics requires the identification of the correct scaffolds as well as of key residues to mimic. Towards this end, non-peptide mimetics containing the scaffolds of dendritic, 8-hydroxy-2-(1H)-quinolinone and the 5-hydroxymethyl resorcinol and the residues Leu, Arg, and Tyr, which matched the pharmacophore found in the conotoxin, were developed as MVIIA mimetics and show promissory biological activities against Ca_V_2.2. Conotoxins remain an attractive option for the development of new therapeutic strategies using bioactive mimetics against chronic pain. Nevertheless, additional work involving both experimental and theoretical approaches are needed to unravel at the structural level the mechanisms modulating the protein targets of these peptides.

## Figures and Tables

**Table 1:** Classification of conotoxins and their molecular targets ^1^.

Conotoxin Family	Molecular Target	Reference
α (*alpha*)	Nicotinic acetylcholine receptors (nAChR)	[3]
γ (g*amma*)	Neuronal pacemaker cation currents (inward cation current)	[4]
δ (delta)	Voltage-gated sodium (Na^+^) channels	[5]
ε (*epsilon*)	Presynaptic calcium (Ca^2+^) channels or G protein-coupled presynaptic receptors	[6]
ι (*iota*)	Voltage-gated sodium (Na^+^) channels	[7]
κ (*kappa*)	Voltage-gated potassium (K^+^) channels	[8]
μ (*mu*)	Voltage-gated sodium (Na^+^)channels	[9]
ρ (*rho*)	Alpha1-adrenoceptors (GPCR)	[10]
σ (*sigma*)	Serotonin-gated ion channels 5-HT3	[11]
τ (*tau*)	Somatostatin receptor	[12]
χ (*chi*)	Neuronal noradrenaline transporter	[10]
ω (*omega*)	Voltage-gated calcium (Ca_V_) channels	[13]

^1^ Taken and adapted from www.conoserver.org [14,15].

**Table 2:** Types of calcium channels in vertebrates [19,21].

Ca Channel	Human Gene Name	Voltage Activation	α_1_ Subunit	Ca Current
Ca_V_1.1–1.4	*CACNA1S; CACNA1C; CACNA1D; CACNA1F*	HVA	α_1S, C, D, F_	L
Ca_V_2.1	*CACNA1A*	HVA	α_1A_	P/Q
Ca_V_2.2	*CACNA1B*	HVA	α_1B_	N
Ca_V_2.3	*CACNA1E*	HVA	α_1E_	R
Ca_V_3.1–3.3	*CACNA1G; CACNA1H; CACNA1I*	LVA	α_1G, H, I._	T

HVA: High Voltage activated; LVA: Low Voltage activated.

**Table 3:** ω-Conotoxins from *Conus* species and their targets.

Specie Conus	ω-Conotoxin	Alternative Names	Target	Organism	IC_50_	Reference
*C. geographus*	GVIA	G6a, SNX-124,	Ca_V_2.1	*R. norvegicus*	1.05 μM ^1^	[57]
			Ca_V_2.2	*R. norvegicus*	2.02 pM ^1^	[62]
	GVIB		?			[34]
	GVIC		?			[34]
	GVIIA	SNX-178	Ca_V_2.2	*R. norvegicus*	22.9 nM ^1^	[64]
	GVIIB		?			[34]
*C. magus*	MVIIA	M7a, SNX-111, Ziconotide, Prialt^®^	Ca_V_2.1	*R. norvegicus*	156 nM ^1^	[62]
			Ca_V_2.2	*H. sapiens*	7.96 nM ^2^	[59]
	MVIIB	SNX-159	Ca_V_2.2	*R. norvegicus*	101 pM ^1^	[65]
	MVIIC	M7c, SNX-230	Ca_V_2.1	*R. norvegicus*	600 pM ^1^	[57]
			Ca_V_2.2	*R. norvegicus*	7.0 nM ^1^	[57]
	MVIID	SNX-238	?			[52]
*C. striatus*	SVIA	S6a, SNX-157	Ca_V_2.2	*R. norvegicus*	1.46 μM ^1^	[65]
	SVIB	S6b, SNX-183	Ca_V_2.1			[38]
			Ca_V_2.2	*R. norvegicus*	1.09 nM ^1^	[65]
	SO-3		Ca_V_2.2		160 nM ^2^	[45]
	SO-4		?			[66]
	SO-5		?			[66]
*C. catus*	CVIA	C6a, catus-C1b	Ca_V_2.1	*R. norvegicus*	850 nM ^1^	[57]
			Ca_V_2.2	*R. norvegicus*	560 pM ^1^	[57]
	CVIB	C6b	Ca_V_2.1 Ca_V_2.2	*R. norvegicus* *R. norvegicus*	11 nM ^1^ 7.7 nM ^1^ 12 nM ^2^	[57] [57] [67]
	CVIC	C6c	Ca_V_2.1	*R. norvegicus*	31 nM ^1^	[57]
			Ca_V_2.2	*R. norvegicus*	7.6 nM ^1^	[57]
	CVID	AM-336, AM336, leconotide	Ca_V_2.1	*R. norvegicus*	55 μM ^1^	[57]
			Ca_V_2.2	*R. norvegicus*	70 pM ^1^	[57]
	CVIE		Ca_V_2.2	*R. norvegicus*	2.6 nM ^2^ 0.12 nM ^2^	[67]
	CVIF	C6f	Ca_V_1.2	*R. norvegicus*	>3 μM ^2^	[67]
			Ca_V_1.3	*R. norvegicus*	>3 μM ^2^	[67]
			Ca_V_2.2	*R. norvegicus*	19.9 nM/	[67]
					0.1 nM ^2^	[67]
			Ca_V_2.3	*R. norvegicus*	>3 μM ^2^	[67]
*C. fulmen*	FVIA		Ca_V_2.2	*H. sapiens*	11.5 nM ^2^	[59]
*C. radiatus*	RVIA	R6a	Ca_V_2.2	*R. norvegicus*	229 nM ^1^	[39]
*C. textile*	TxVII		L-type			[60]
*C. consors*	CnVIIA	Cn7a, CnVIIH	Ca_V_2.1	*R. norvegicus*	179 nM ^1^	[62]
			Ca_V_2.2	*R. norvegicus*	2.3–3.7 pM ^1^	[62]
	CnVIIB	CnVIIG	?			[63]
	CnVIIC	CnVIIE	?			[63]
*C. pennaceus*	PnVIA	Pn6a	?	*Lymnaea stagnalis*	~5 μM^2^	[68]
	PnVIB	Pn6b	?	*Lymnaea stagnalis*	~5 μM^2^	[68]
*C. tulipa*	TVIA	SNX-185	Ca_V_2.2	*R. norvegicus*	228 pM ^1^	[65]

^1^ Binding/competition assay; ^2^ Electrophysiological measurements.

## References

[1] Becker S., Terlau H. (2008). Toxins from cone snails: Properties, applications and biotechnological production. Appl. Microbiol. Biotechnol..

[2] Terlau H., Olivera B.M. (2004). Conus venoms: A rich source of novel ion channel-targeted peptides. Physiol. Rev..

[3] Gray W.R., Luque A., Olivera B.M., Barrett J., Cruz L.J. (1981). Peptide toxins from Conus geographus venom. J. Biol. Chem..

[4] Fainzilber M., Nakamura T., Lodder J.C., Zlotkin E., Kits K.S., Burlingame A.L. (1998). γ-Conotoxin-PnVIIA, a γ-carboxyglutamate-containing peptide agonist of neuronal pacemaker cation currents. Biochemistry.

[5] Fainzilber M., Gordon D., Hasson A., Spira M.E., Zlotkin E. (1991). Mollusc-specific toxins from the venom of Conus textile neovicarius. FEBS J..

[6] Rigby A.C., Lucas-Meunier E., Kalume D.E., Czerwiec E., Hambe B., Dahlqvist I., Fossier P., Baux G., Roepstorff P., Baleja J.D. (1999). A conotoxin from Conus textile with unusual posttranslational modifications reduces presynaptic Ca^2+^ influx. Proc. Natl. Acad. Sci. USA.

[7] Buczek O., Wei D., Babon J.J., Yang X., Fiedler B., Chen P., Yoshikami D., Olivera B.M., Bulaj G., Norton R.S. (2007). Structure and sodium channel activity of an excitatory I1-superfamily conotoxin. Biochemistry.

[8] Terlau H., Shon K.-J., Grilley M., Stocker M., Stühmer W., Olivera B.M. (1996). Strategy for rapid immobilization of prey by a fish-hunting marine snail. Nature.

[9] Cruz L.J., Gray W.R., Olivera B.M., Zeikus R.D., Kerr L., Yoshikami D., Moczydlowski E. (1985). Conus geographus toxins that discriminate between neuronal and muscle sodium channels. J. Biol. Chem..

[10] Sharpe I.A., Gehrmann J., Loughnan M.L., Thomas L., Adams D.A., Atkins A., Palant E., Craik D.J., Adams D.J., Alewood P.F. (2001). Two new classes of conopeptides inhibit the α1-adrenoceptor and noradrenaline transporter. Nat. Neurosci..

[11] England L.J., Imperial J., Jacobsen R., Craig A.G., Gulyas J., Akhtar M., Rivier J., Julius D., Olivera B.M. (1998). Inactivation of a serotonin-gated ion channel by a polypeptide toxin from marine snails. Science.

[12] Petrel C., Hocking H.G., Reynaud M., Upert G., Favreau P., Biass D., Paolini-Bertrand M., Peigneur S., Tytgat J., Gilles N. (2013). Identification, structural and pharmacological characterization of τ-CnVA, a conopeptide that selectively interacts with somatostatin sst 3 receptor. Biochem. Pharmacol..

[13] Kerr L.M., Yoshikami D. (1984). A venom peptide with a novel presynaptic blocking action. Nature.

[14] Kaas Q., Westermann J.-C., Halai R., Wang C.K.L., Craik D.J. (2007). ConoServer, a database for conopeptide sequences and structures. Bioinformatics.

[15] Kaas Q., Yu R., Jin A.-H., Dutertre S., Craik D.J. (2011). ConoServer: Updated content, knowledge, and discovery tools in the conopeptide database. Nucleic Acids Res..

[16] Olivera B.M., Rivier J., Scott J.K., Hillyard D.R., Cruz L.J. (1991). Conotoxins. J. Biol. Chem..

[17] Adams D.J., Berecki G. (2013). Mechanisms of conotoxin inhibition of N-type (Cav2.2) calcium channels. Biochim. Biophys. Acta Biomembr..

[18] Bourinet E., Zamponi G.W. (2016). Block of voltage-gated calcium channels by peptide toxins. Neuropharmacology.

[19] Hille B. (2001). Ion Channels of Excitable Membranes.

[20] Dolphin A.C. (2016). Voltage-gated calcium channels and their auxiliary subunits: Physiology and pathophysiology and pharmacology. J. Physiol..

[21] Schroeder C.I., Lewis R.J. (2006). ω-conotoxins GVIA, MVIIA and CVID: SAR and clinical potential. Mar. Drugs.

[22] Clapham D.E. (1995). Calcium signaling. Cell.

[23] Zamponi G.W., Striessnig J., Koschak A., Dolphin A.C. (2015). The physiology, pathology, and pharmacology of voltage-gated calcium channels and their future therapeutic potential. Pharmacol. Rev..

[24] Albillos A., Neher E., Moser T. (2000). R-Type Ca^2+^ channels are coupled to the rapid component of secretion in mouse adrenal slice chromaffin cells. J. Neurosci..

[25] Loane D.J., Lima P.A., Marrion N. (2007). V Co-assembly of N-type Ca^2+^ and BK channels underlies functional coupling in rat brain. J. Cell Sci..

[26] Jun K., Piedras-Renteria E.S., Smith S.M., Wheeler D.B., Lee S.B., Lee T.G., Chin H., Adams M.E., Scheller R.H., Tsien R.W. (1999). Ablation of P/Q-type Ca^2+^ channel currents, altered synaptic transmission, and progressive ataxia in mice lacking the α1A-subunit. Proc. Natl. Acad. Sci. USA.

[27] Saegusa H., Kurihara T., Zong S., Kazuno A., Matsuda Y., Nonaka T., Han W., Toriyama H., Tanabe T. (2001). Suppression of inflammatory and neuropathic pain symptoms in mice lacking the N-type Ca^2+^ channel. EMBO J..

[28] Bourinet E., Altier C., Hildebrand M.E., Trang T., Salter M.W., Zamponi G.W. (2014). Calcium-permeable ion channels in pain signaling. Physiol. Rev..

[29] Schroeder C.I., Rash L.D., Vila-Farrés X., Rosengren K.J., Mobli M., King G.F., Alewood P.F., Craik D.J., Durek T. (2014). Chemical Synthesis, 3D Structure, and ASIC Binding Site of the Toxin Mambalgin-2. Angew. Chem. Int. Ed..

[30] Pallaghy P.K., Norton R.S., Nielsen K.J., Craik D.J. (1994). A common structural motif incorporating a cystine knot and a triple-stranded β-sheet in toxic and inhibitory polypeptides. Protein Sci..

[31] Sigrist C.J.A., Cerutti L., Hulo N., Gattiker A., Falquet L., Pagni M., Bairoch A., Bucher P. (2002). PROSITE: A documented database using patterns and profiles as motif descriptors. Brief. Bioinform..

[32] Sigrist C.J.A., De Castro E., Cerutti L., Cuche B.A., Hulo N., Bridge A., Bougueleret L., Xenarios I. (2012). New and continuing developments at PROSITE. Nucleic Acids Res..

[33] Ellinor P.T., Zhang J.F., Horne W.A., Tsien R.W. (1994). Structural determinants of the blockade of N-type calcium channels by a peptide neurotoxin. Nature.

[34] Olivera B.M., Gray W.R., Zeikus R., McIntosh J.M., Varga J., Rivier J., De Santos V., Cruz L.J. (1985). Peptide neurotoxins from fish-hunting cone snails. Science.

[35] McCleskey E.W., Fox A.P., Feldman D.H., Cruz L.J., Olivera B.M., Tsien R.W., Yoshikami D. (1987). Omega-conotoxin: Direct and persistent blockade of specific types of calcium channels in neurons but not muscle. Proc. Natl. Acad. Sci. USA.

[36] Regan L.J., Sah D.W.Y., Bean B.P. (1991). Ca^2+^ channels in rat central and peripheral neurons: High-threshold current resistant to dihydropyridine blockers and ω-conotoxin. Neuron.

[37] Olivera B.M., Cruz L.J., De Santos V., LeCheminant G., Griffin D., Zeikus R., McIntosh J.M., Galyean R., Varga J. (1987). Neuronal calcium channel antagonists. Discrimination between calcium channel subtypes using omega.-conotoxin from Conus magus venom. Biochemistry.

[38] Ramilo C.A., Zafaralla G.C., Nadasdi L., Hammerland L.G., Yoshikami D., Gray W.R., Kristipati R., Ramachandran J., Miljanich G., Olivera B.M. (1992). Novel alpha- and omega-conotoxins from Conus striatus venom. Biochemistry.

[39] Miljanich G.P., Bitner R.S., Bowersox S.S., Fox J.A., Valentino K.L., Yamashiro D.H. (1991). Method of Treating Ischemia-Related Neuronal Damage. U.S. Patent.

[40] Pallaghy P.K., Norton R.S. (1999). Refined solution structure of ω-conotoxin GVIA: Implications for calcium channel binding. J. Pept. Res..

[41] Mould J., Yasuda T., Schroeder C.I., Beedle A.M., Doering C.J., Zamponi G.W., Adams D.J., Lewis R.J. (2004). The α2δ auxiliary subunit reduces affinity of ω-conotoxins for recombinant N-type (Cav2.2) calcium channels. J. Biol. Chem..

[42] Davis J.H., Bradley E.K., Miljanich G.P., Nadasdi L., Ramachandran J., Basus V.J. (1993). Solution structure of. omega.-conotoxin GVIA using 2-D NMR spectroscopy and relaxation matrix analysis. Biochemistry.

[43] Wang Y.-X., Pettus M., Gao D., Phillips C., Bowersox S.S. (2000). Effects of intrathecal administration of ziconotide, a selective neuronal N-type calcium channel blocker, on mechanical allodynia and heat hyperalgesia in a rat model of postoperative pain. Pain.

[44] Miljanich G.P. (2004). Ziconotide: Neuronal calcium channel blocker for treating severe chronic pain. Curr. Med. Chem..

[45] Wang F., Yan Z., Liu Z., Wang S., Wu Q., Yu S., Ding J., Dai Q. (2016). Molecular basis of toxicity of N-type calcium channel inhibitor MVIIA. Neuropharmacology.

[46] Skov M.J., Beck J.C., de Kater A.W., Shopp G.M. (2007). Nonclinical safety of ziconotide: An intrathecal analgesic of a new pharmaceutical class. Int. J. Toxicol..

[47] McGivern J.G. (2007). Ziconotide: A review of its pharmacology and use in the treatment of pain. Neuropsychiatr. Dis. Treat..

[48] Kohno T., Kim J.I., Kobayashi K., Kodera Y., Maeda T., Sato K. (1995). Three-Dimensional Structure in Solution of the Calcium Channel Blocker omega.-Conotoxin MVIIA. Biochemistry.

[49] Nielsen K.J., Thomas L., Lewis R.J., Alewood P.F., Craik D.J. (1996). A consensus structure for ω-conotoxins with different selectivities for voltage-sensitive calcium channel subtypes: Comparison of MVIIA, SVIB and SNX-202. J. Mol. Biol..

[50] Adams D.J., Smith A.B., Schroeder C.I., Yasuda T., Lewis R.J. (2003). ω-conotoxin CVID inhibits a pharmacologically distinct voltage-sensitive calcium channel associated with transmitter release from preganglionic nerve terminals. J. Biol. Chem..

[51] Atkinson R.A., Kieffer B., Dejaegere A., Sirockin F., Lefèvre J.-F. (2000). Structural and dynamic characterization of ω-conotoxin MVIIA: The binding loop exhibits slow conformational exchange. Biochemistry.

[52] Monje V.D., Haack J.A., Naisbitt S.R., Miljanich G., Ramachandran J., Nasdasdi L., Olivera B.M., Hillyard D.R., Gray W.R. (1993). A new Conus peptide ligand for Ca channel subtypes. Neuropharmacology.

[53] Farr-Jones S., Miljanich G.P., Nadasdi L., Ramachandran J., Basus V.J. (1995). Solution structure of ω-conotoxin MVIIC, a high affinity ligand of P-type calcium channels, using1H NMR spectroscopy and complete relaxation matrix analysis. J. Mol. Biol..

[54] Nielsen K.J., Adams D., Thomas L., Bond T., Alewood P.F., Craik D.J., Lewis R.J. (1999). Structure-activity relationships of ω-conotoxins MVIIA, MVIIC and 14 loop splice hybrids at N and P/Q-type calcium channels. J. Mol. Biol..

[55] Wen L., Yang S., Qiao H., Liu Z., Zhou W., Zhang Y., Huang P. (2005). SO-3, a new O-superfamily conopeptide derived from Conus striatus, selectively inhibits N-type calcium currents in cultured hippocampal neurons. Br. J. Pharmacol..

[56] Yan Y., Tu G., Luo X., Dai Q., Huang P., Zhang R. (2003). Three-dimensional solution structure of ω-conotoxin SO_3_ determined by1H NMR. Chin. Sci. Bull..

[57] Lewis R.J., Nielsen K.J., Craik D.J., Loughnan M.L., Adams D.A., Sharpe I.A., Luchian T., Adams D.J., Bond T., Thomas L. (2000). Novel ω-conotoxins from Conus catus discriminate among neuronal calcium channel subtypes. J. Biol. Chem..

[58] Schroeder C.I., Doering C.J., Zamponi G.W., Lewis R.J. (2006). N-type calcium channel blockers: Novel therapeutics for the treatment of pain. Med. Chem..

[59] Lee S., Kim Y., Back S.K., Choi H.-W., Lee J.Y., Jung H.H., Ryu J.H., Suh H.-W., Na H.S., Kim H.J. (2010). Analgesic effect of highly reversible ω-conotoxin FVIA on N type Ca^2+^ channels. Mol. Pain.

[60] Fainzilber M., Lodder J.C., van der Schors R.C., Li K.W., Yu Z., Burlingame A.L., Geraerts W.P.M., Kits K.S. (1996). A novel hydrophobic omega-conotoxin blocks molluscan dihydropyridine-sensitive calcium channels. Biochemistry.

[61] Kobayashi K., Sasaki T., Sato K., Kohno T. (2000). Three-dimensional solution structure of ω-conotoxin TxVII, an L-type calcium channel blocker. Biochemistry.

[62] Favreau P., Gilles N., Lamthanh H., Bournaud R., Shimahara T., Bouet F., Laboute P., Letourneux Y., Ménez A., Molgó J. (2001). A new ω-conotoxin that targets N-type voltage-sensitive calcium channels with unusual specificity. Biochemistry.

[63] Violette A., Biass D., Dutertre S., Koua D., Piquemal D., Pierrat F., Stöcklin R., Favreau P. (2012). Large-scale discovery of conopeptides and conoproteins in the injectable venom of a fish-hunting cone snail using a combined proteomic and transcriptomic approach. J. Proteom..

[64] Miljanich G.P., Bitner R.S., Bowersox S.S., Fox J.A., Valentino K.L., Yamashiro D.H., Tsubokawa M. (1993). Screening Method for Neuroprotective Compounds. U.S. Patent.

[65] Miljanich G.P., Bowersox S.S., Fox J.A., Valentino K.L., Bitner R.S., Yamashiro D.H. (1993). Compositions for Delayed Treatment of Ischemia-Related Neuronal Damage. WO Patent.

[66] Bai-Song L., Fang Y., Dong Z., Pei-Tang H., Cui-Fen H. (1999). Conopeptides from Conus striatus and Conus textile by cDNA cloning. Peptides.

[67] Berecki G., Motin L., Haythornthwaite A., Vink S., Bansal P., Drinkwater R., Wang C.I., Moretta M., Lewis R.J., Alewood P.F. (2010). Analgesic ω-conotoxins CVIE and CVIF selectively and voltage-dependently block recombinant and native N-type calcium channels. Mol. Pharmacol..

[68] Kits K.S., Lodder J.C., Van Der Schors R.C., Li K.W., Geraerts W.P.M., Fainzilber M. (1996). Novel ω-Conotoxins Block Dihydropyridine-Insensitive High Voltage-Activated Calcium Channels in Molluscan Neurons. J. Neurochem..

[69] Feng Z.-P., Hamid J., Doering C., Bosey G.M., Snutch T.P., Zamponi G.W. (2001). Residue Gly1326 of the N-type calcium channel α1B subunit controls reversibility of ω-conotoxin GVIA and MVIIA block. J. Biol. Chem..

[70] Schroeder C.I., Smythe M.L., Lewis R.J. (2004). Development of small molecules that mimic the binding of ω-conotoxins at the N-type voltage-gated calcium channel. Mol. Divers..

[71] Baell J.B., Duggan P.J., Forsyth S.A., Lewis R.J., Lok Y.P., Schroeder C.I. (2004). Synthesis and biological evaluation of nonpeptide mimetics of ω-conotoxin GVIA. Bioorg. Med. Chem..

[72] Nadasdi L., Yamashiro D., Chung D., Tarczy-Hornoch K., Adriaenssens P., Ramachandran J. (1995). Structure-Activity Analysis of a Conus Peptide Blocker of N-Type Neuronal Calcium Channels. Biochemistry.

[73] Menzler S., Bikker J.A., Horwell D.C. (1998). Synthesis of a non-peptide analogue of omega-conotoxin MVIIA. Tetrahedron Lett..

[74] Menzler S., Bikker J.A., Suman-Chauhan N., Horwell D.C. (2000). Design and biological evaluation of non-peptide analogues of omega-conotoxin MVIIA. Bioorg. Med. Chem. Lett..

[75] Guo Z.-X., Cammidge A.N., Horwell D.C. (2000). Dendroid peptide structural mimetics of ω-conotoxin MVIIA based on a 2 (1H)-quinolinone core. Tetrahedron.

[76] Duggan P.J., Tuck K.L. (2015). Bioactive mimetics of conotoxins and other venom peptides. Toxins.

[77] Layer R.T., Mcintosh J.M. (2006). Conotoxins: Therapeutic Potential and Application. Mar. Drugs.

[78] Yeager R.E., Yoshikami D., Rivier J., Cruz L.J., Miljanich G.P. (1987). Transmitter release from presynaptic terminals of electric organ: Inhibition by the calcium channel antagonist omega Conus toxin. J. Neurosci..

[79] Malmberg A.B., Yaksh T.L. (1995). Effect of continuous intrathecal infusion of ω-conopeptides, N-type calcium-channel blockers, on behavior and antinociception in the formalin and hot-plate tests in rats. Pain.

[80] Sluka K.A. (1998). Blockade of N-and P/Q-type calcium channels reduces the secondary heat hyperalgesia induced by acute inflammation. J. Pharmacol. Exp. Ther..

[81] Xiao W.H., Bennett G.J. (1995). Synthetic omega-conopeptides applied to the site of nerve injury suppress neuropathic pains in rats. J. Pharmacol. Exp. Ther..

[82] Wang Y.-X., Gao D., Pettus M., Phillips C., Bowersox S.S. (2000). Interactions of intrathecally administered ziconotide, a selective blocker of neuronal N-type voltage-sensitive calcium channels, with morphine on nociception in rats. Pain.

[83] Hannon H.E., Atchison W.D. (2013). Omega-conotoxins as experimental tools and therapeutics in pain management. Mar. Drugs.

[84] Kim J.I., Takahashi M., Ogura A., Kohno T., Kudo Y., Sato K. (1994). Hydroxyl group of Tyr13 is essential for the activity of omega-conotoxin GVIA, a peptide toxin for N-type calcium channel. J. Biol. Chem..

[85] Lew M.J., Flinn J.P., Pallaghy P.K., Murphy R., Whorlow S.L., Wright C.E., Norton R.S., Angus J.A. (1997). Structure-function relationships of ω-conotoxin GVIA Synthesis, structure, calcium channel binding, and functional assay of alanine-substituted analogues. J. Biol. Chem..

